# Supplementary material for: Epigenetic modulation rescues neurodevelopmental deficits in Syngap1+/− mice
Source: Aging Cell. 2025 Jan 29;24(3):e14408. doi: 10.1111/acel.14408 (PMC11896221; doi:10.1111/acel.14408)
Supplement: Supplementary file 1 — Appendix S1: Supporting Information. [file ACEL-24-e14408-s001.pdf]

Supplementary Materials

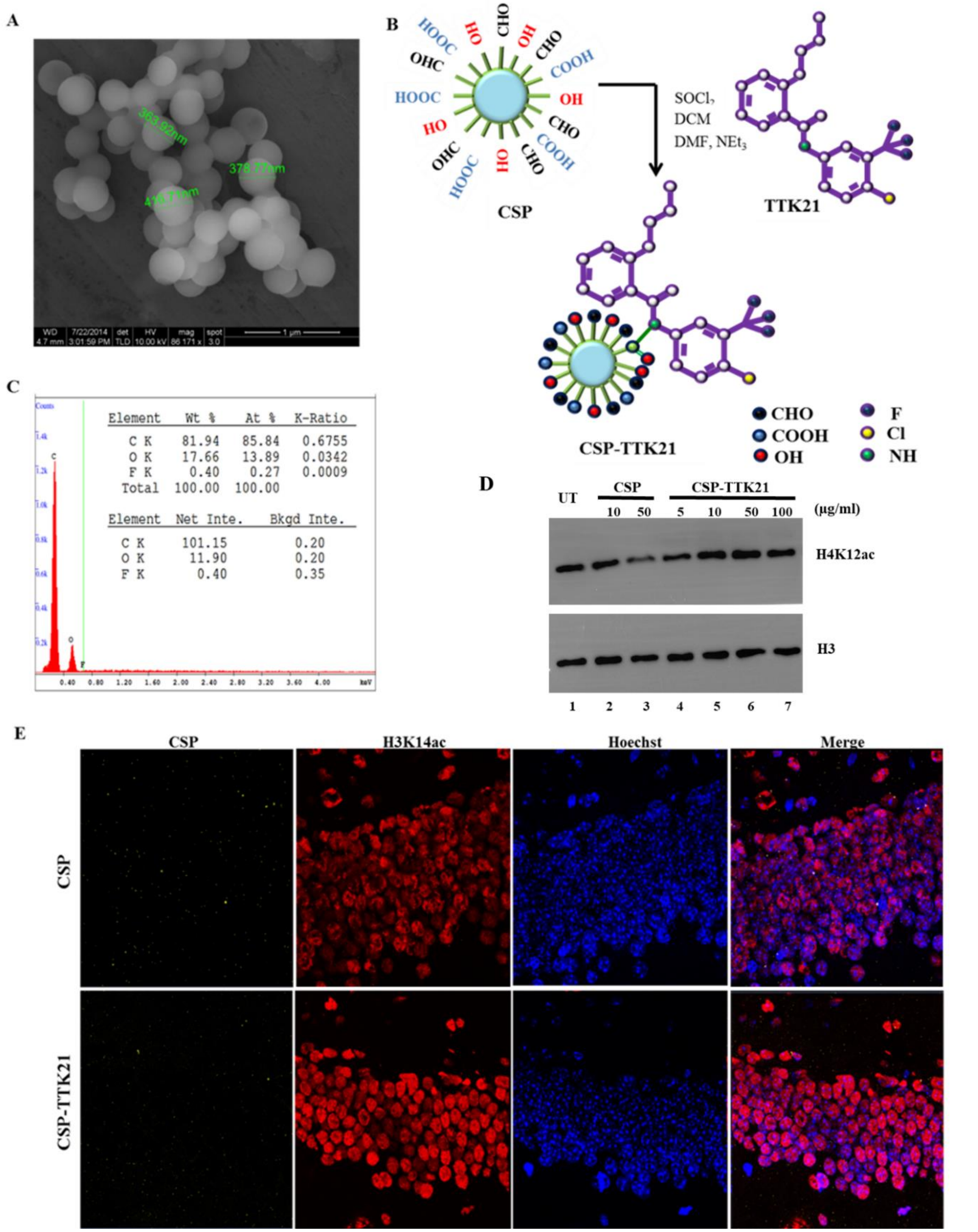

Fig. S1. Synthesis and characterization of CSP-TTK21. (A) FESEM image of CSP, (B) Schematic showing the synthesis of CSP-TTK21, (C) fluorine upon EDX analysis of CSP-TTK21 confirming the conjugation of TTK21 with CSP. (D) Immuno-blots showing increased H4K12

acetylation in SHSY-5Y cells upon CSP-TTK21 treatment. (E) Induction of H3K14 acetylation in dorsal hippocampus of mouse brain treated with CSP-TTK21 shows CSP-TTK21 is functionally active. Scale (A) 1µm and (E) 10µm. UT- untreated and CSP-vehicle control.

Fig. S2.

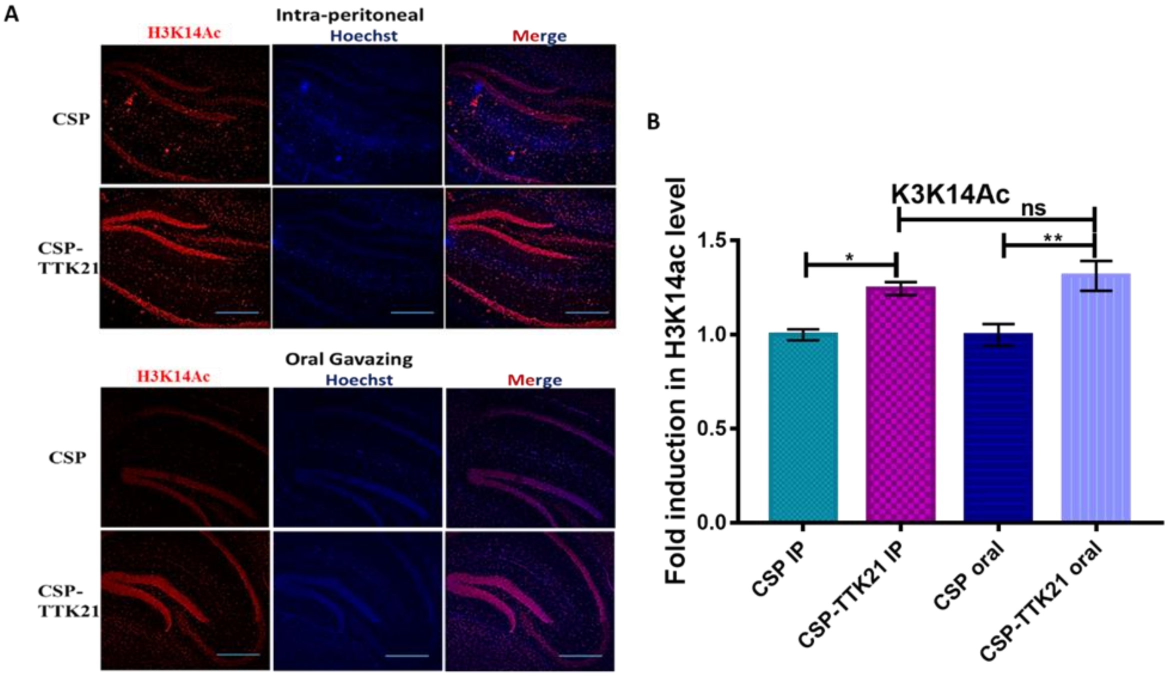

Fig. S2. CSP-TTK21 induces histone acetylation upon oral gavaging. (A) In both cases of IP and Oral administration, acetylation was increased in the hippocampal region compared to CSP-control (n=4). Scale bar 200µm. (B) Quantitation of fold induction in histone acetylation level upon Oral and IP administration of CSP-TTK21 (normalized to CSP). Error bars represent the standard error of mean (SEM). Student's t-test was done for significance test, $*p<0.05$ and $**p<0.01$.

Fig. S3.

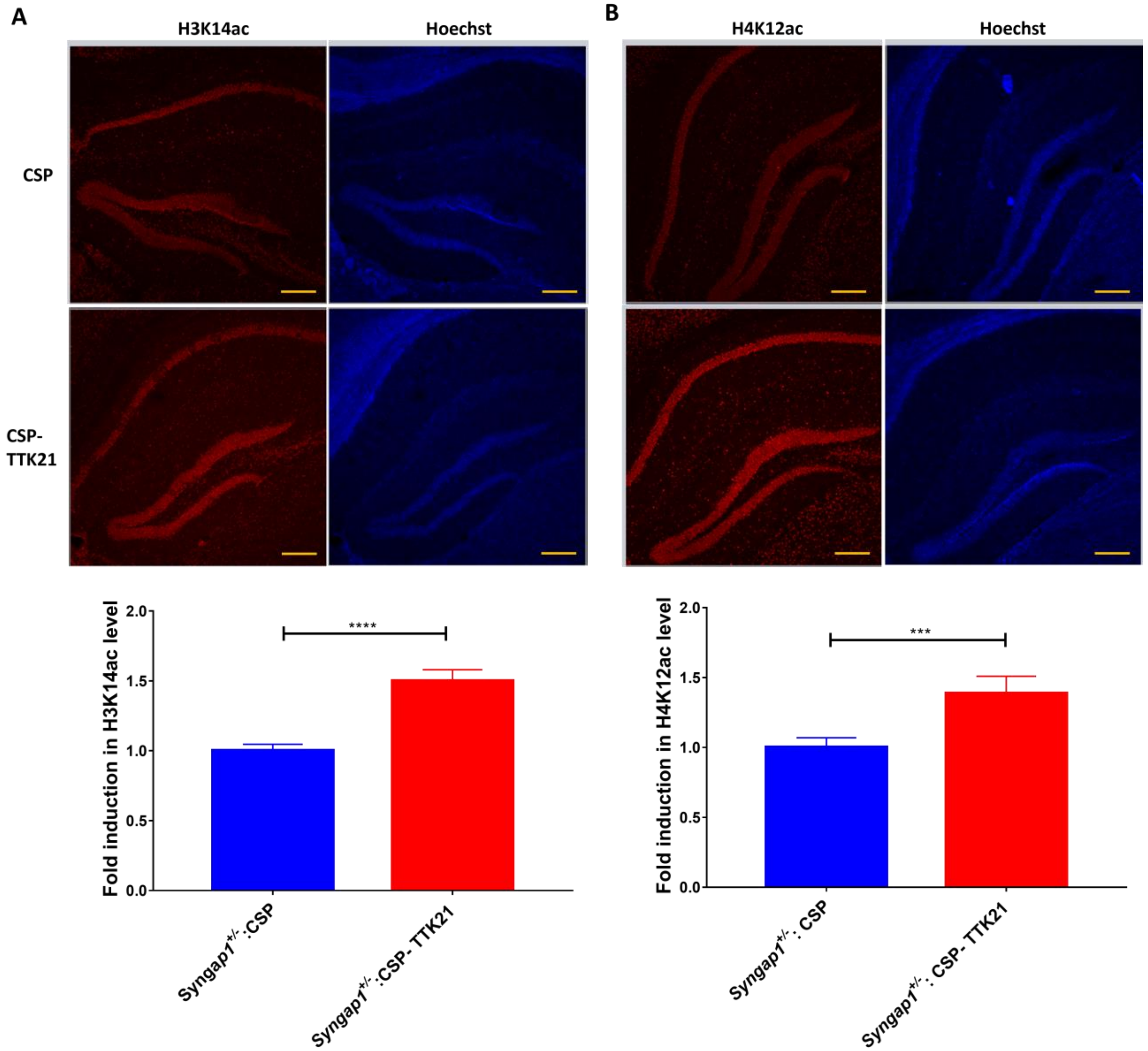

Fig. S3. CSP-TTK21 treatment increased histone acetylation in *Syngap1*$^{+/-}$ mouse. Representative confocal images showing induction of (A) H3K14Ac levels and (B) H4K12Ac levels in the dorsal hippocampus of *Syngap1*$^{+/-}$ mice upon treatment with CSP-TTK21 as compared to CSP vehicle control. Error bars represent the standard error of mean (SEM). Student's t-test was done for significance test, ***$p<0.001$, and ****$p<0.0001$.

Fig. S4.

A

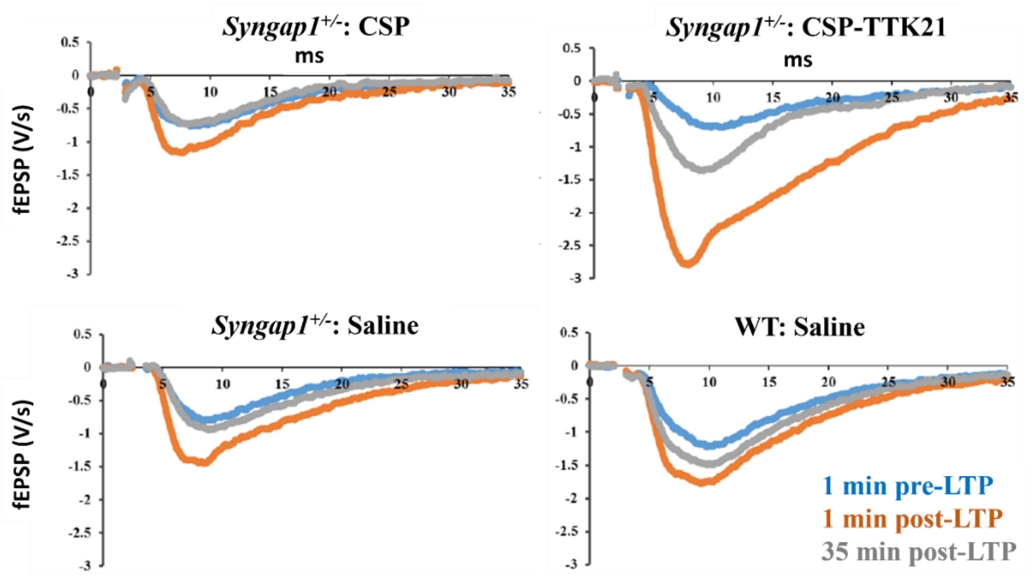

B

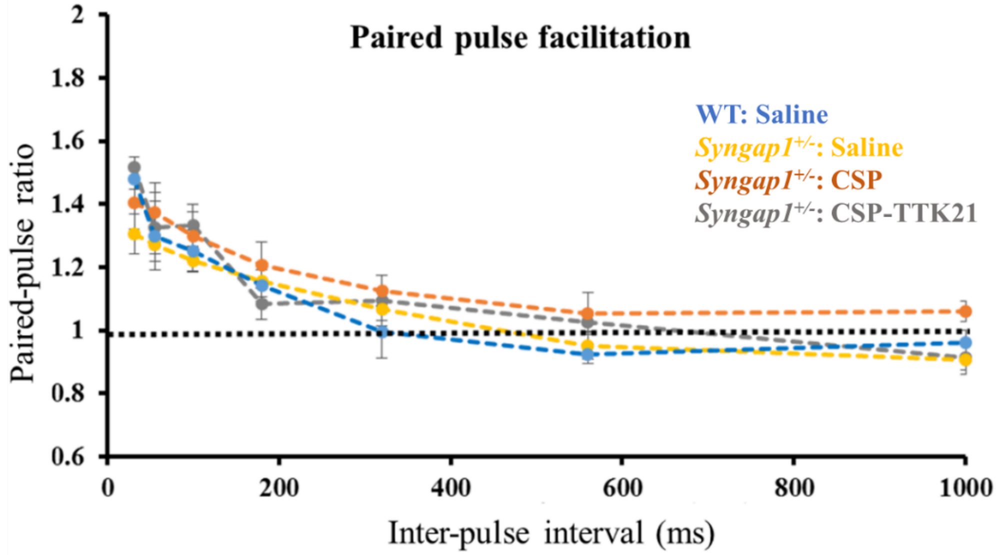

Fig. S4. CSP-TTK21 induces LTP potentiation, not paired-pulse response (PPR). (A) Representative sample traces for each group were taken 1 min before (blue), 1 min after (orange), and 35 min after (grey) LTP induction. (B) Two presynaptic spikes were evoked simultaneously at different time intervals, and the ratio of the two post-synaptic responses (fEPSP2/fEPSP1) was measured and plotted against time intervals. No significant alteration was seen in paired-pulse facilitation, $p>0.06$. Significance was tested by Two-way ANOVA (repeated measures). Error bars represent SEM. n= 4 slices.

Fig. S5.

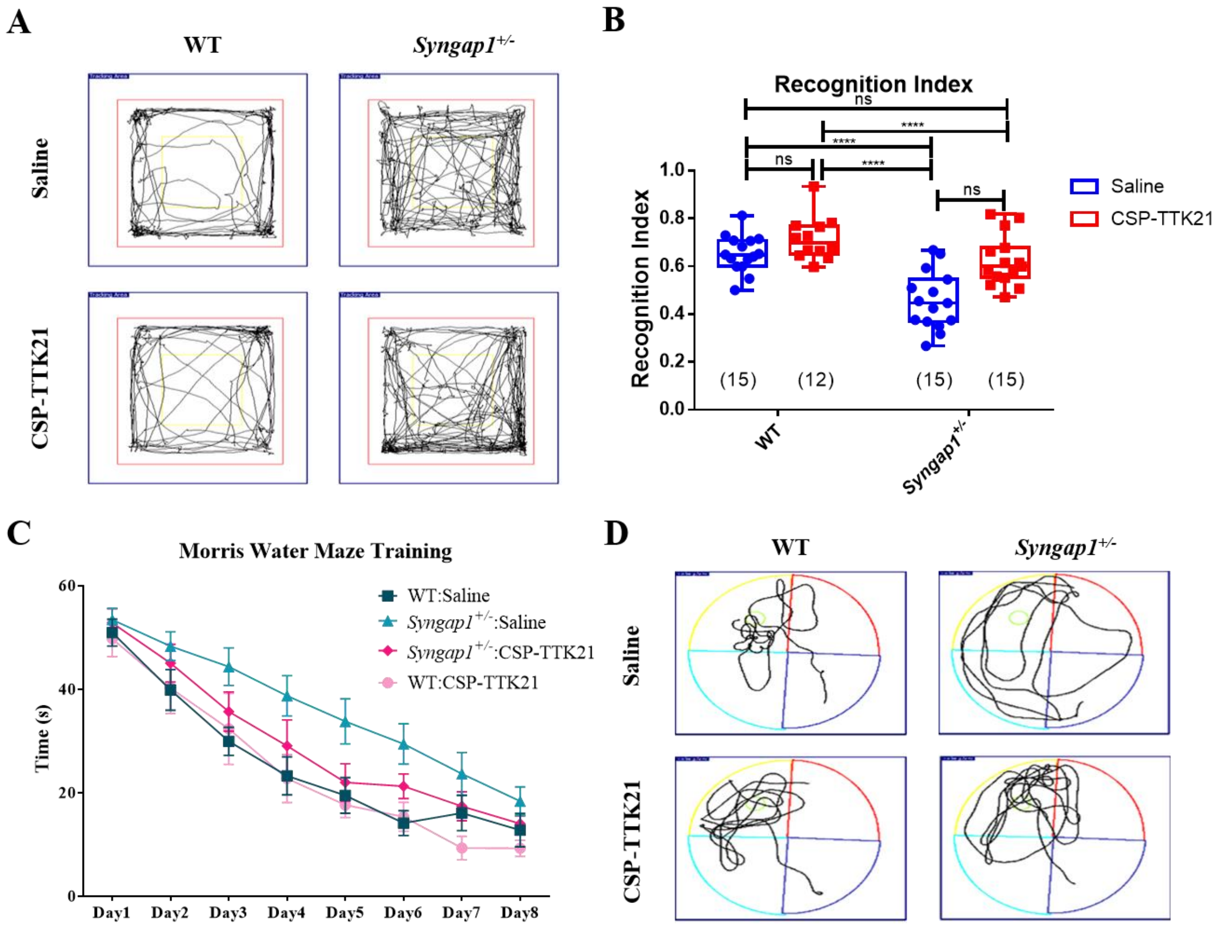

Fig. S5. CSP-TTK21 effects on behavioural measures in *Syngap1*$^{+/-}$ mouse. (A) OFT representative tracks. (B) Recognition index (RI) in Novel object recognition memory, reduced RI in *Syngap1*$^{+/-}$ mice representing impaired object recognition memory. CSP-TTK21 treatment increases RI in *Syngap1*$^{+/-}$ mice. (C) The latency time across different days of MWM training (12-15 mice/group). (B) Representative traces from each group during the probe test depicting localized movement in the target zone (yellow) in CSP-TTK21 treated Syngap1+/- mice as compared to random movement in the *Syngap1*$^{+/-}$:Saline group.

Fig. S6.

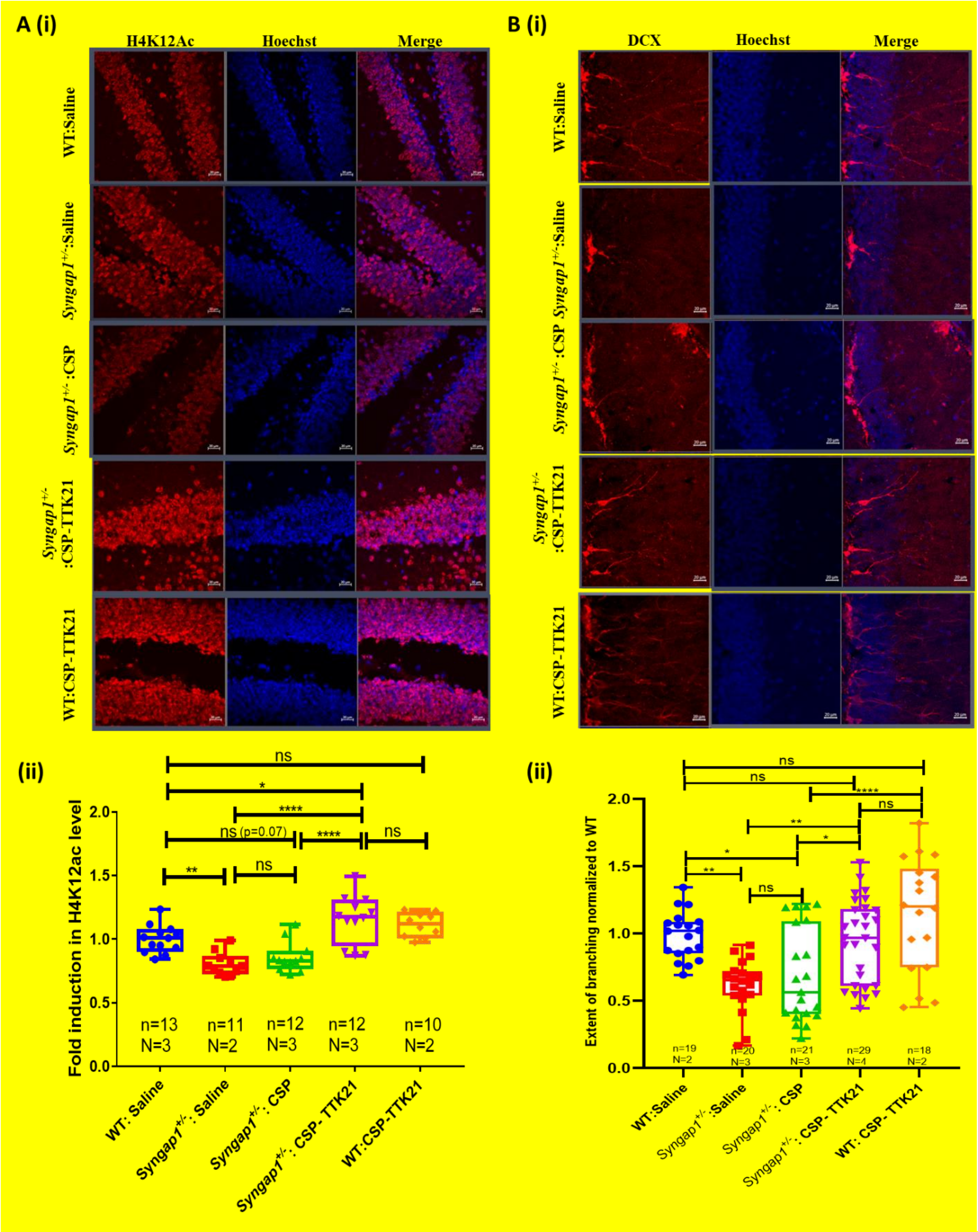

Figure S6. Reversal of acetylation and dendritic branching in animals subjected to behavioral analysis. A) Representative confocal images showing H4K12Ac expression in dorsal hippocampus of the different experimental groups used (*Syngap1*$^{+/-}$:Saline, *Syngap1*$^{+/-}$:CSP, *Syngap1*$^{+/-}$:CSP-TTK21, and WT: CSP-TTK21) depicting reduced levels in *Syngap1*$^{+/-}$ mice

treated with saline or CSP, and restoration to WT levels in CSP-TTK21 treated *Syngap1*$^{+/-}$ mice. CSP-TTK21 treated WT mice also showed increased levels of H4K12Ac as compared to control WT, depicting CSP-TTK21 functional activity. C) Representative confocal images of DCX^{+}-neurons in the subventricular zone of Hippocampus in the treatment groups, depicting reduced dendritic branching in saline or CSP treated *Syngap1*$^{+/-}$ mice, and restoration to WT levels in CSP-TTK21 treated *Syngap1*$^{+/-}$ mice. Scale 20 µm, error bars represent the s.e.m.

Fig S7

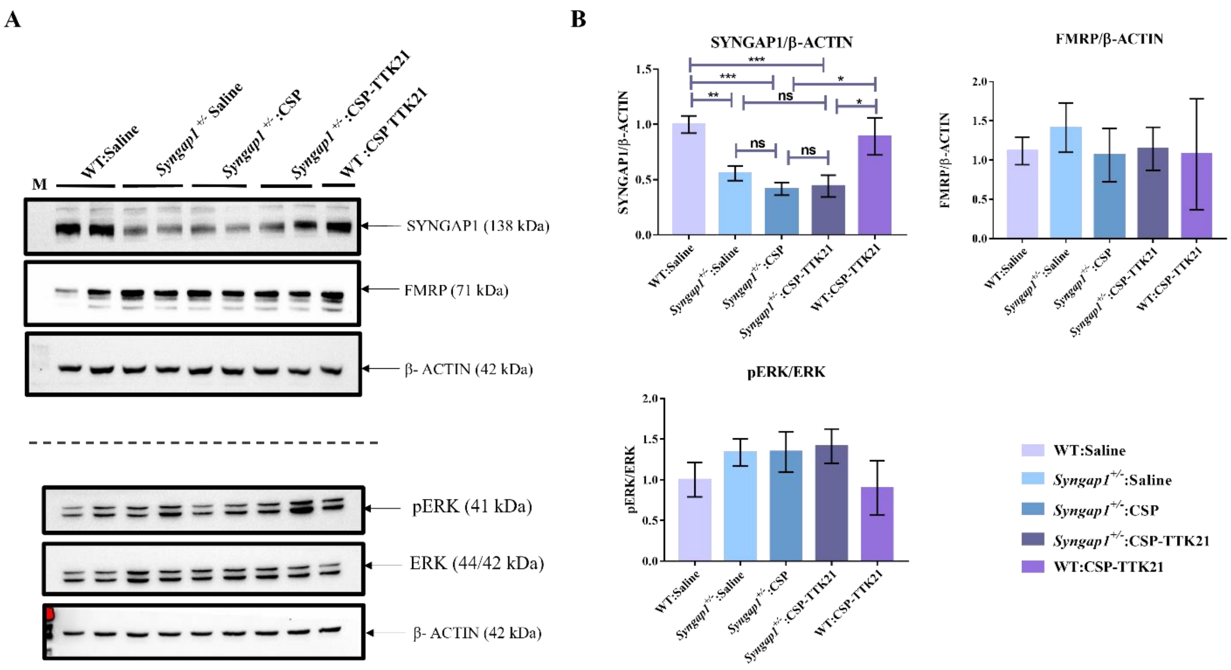

Fig. S7. Western blotting from mice lysate after treatment with CSP or CSP-TTK21. Mice were sacrificed, and tissue lysates were prepared after three days of CSPTTK21 treatment (4-6 mice/group). (A) Representative blot images and (B) Quantifications showing no effect of CSP-TTK21 on SYNGAP1, p-ERK/ERK, and FMRP levels.

Fig. S8.

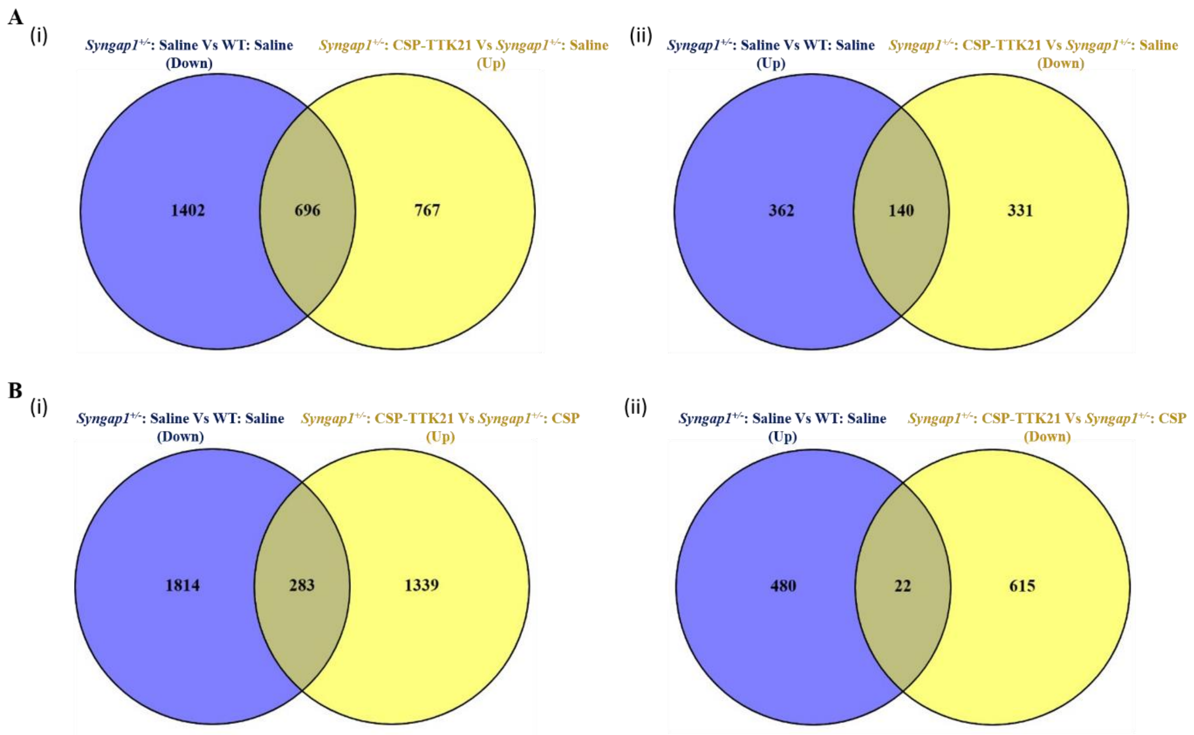

Fig. S8. Venn diagram showing the effect of CSP-TTK21 in restoration of significantly deregulated genes across different treatment conditions. A. i) De down-regulated in *Syngap1*$^{+/-}$ mice (blue) and up-regulated in *Syngap1*$^{+/-}$: CSP-TTK21 mice (yellow)**. (A. ii)** Up-regulated in *Syngap1*$^{+/-}$ mice (blue) and down-regulated in *Syngap1*$^{+/-}$: CSP-TTK21 mice (yellow). The overlap region (yellowish blue) depicts the restoration of DEGs upon CSP-TTK21 treatment. B. i) De down-regulated in *Syngap1*$^{+/-}$ mice (blue) and up-regulated in *Syngap1*$^{+/-}$: CSP-TTK21 mice as compared to CSP vehicle control (yellow)**. (B. ii)** Up-regulated in *Syngap1*$^{+/-}$ mice (blue) and down-regulated in *Syngap1*$^{+/-}$: CSP-TTK21 mice as compared to CSP vehicle control (yellow). The overlap region (yellowish blue) depicts the restoration of DEGs upon CSP-TTK21 treatment.

Fig. S9.

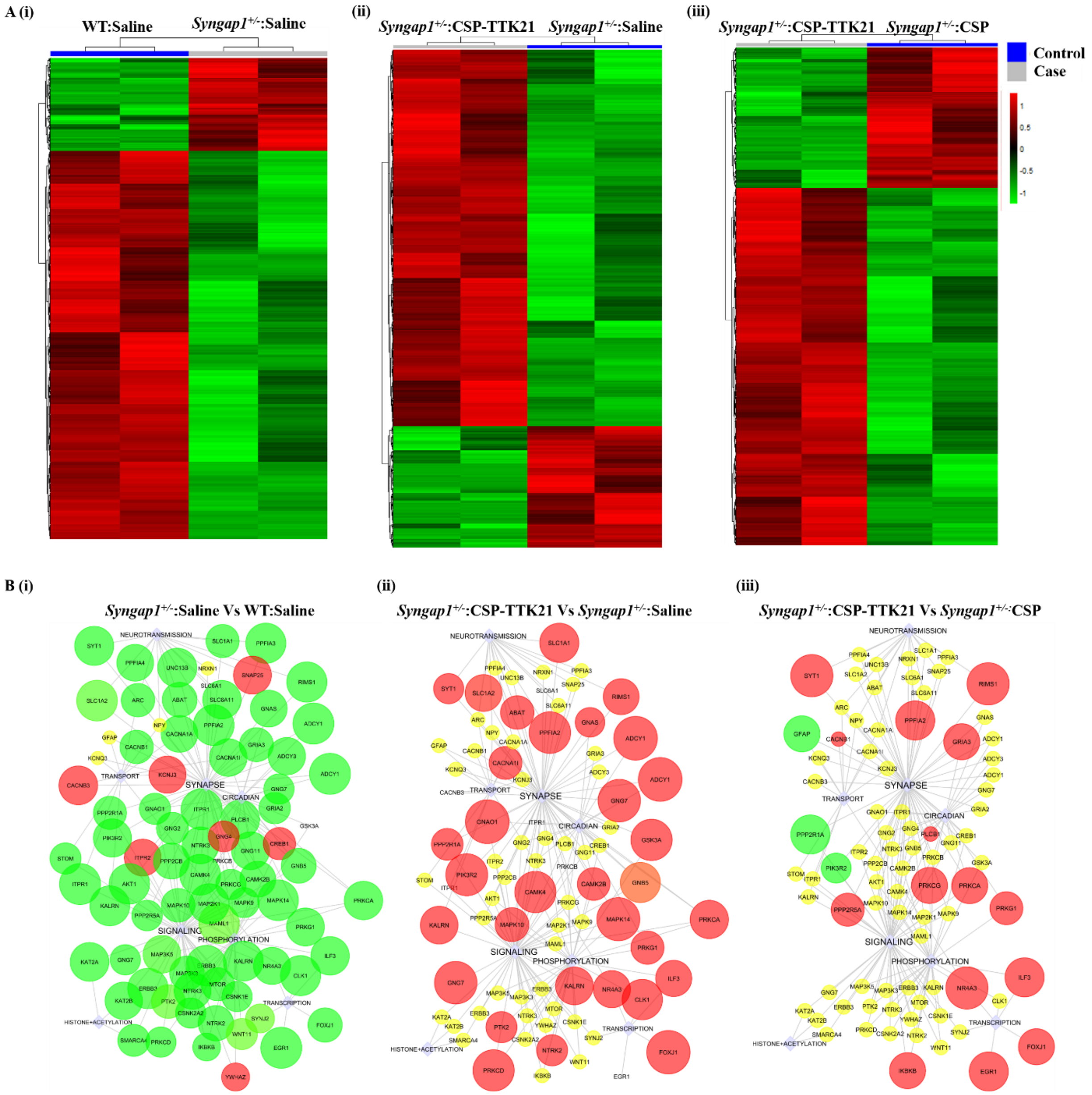

Fig. S9. CSP-TTK21 restores gene expression and signaling pathways that were altered in *Syngap1*$^{+/-}$ mouse. (A) Heatmaps representing the differentially expressed transcripts across various conditions. (i) *Syngap1*$^{+/-}$: Saline Vs WT: Saline, (ii) *Syngap1*$^{+/-}$: CSP-TTK21 Vs *Syngap1*$^{+/-}$: Saline, and (iii) *Syngap1*$^{+/-}$: CSP-TTK21 Vs *Syngap1*$^{+/-}$: CSP. Blue colour represents the control group and grey represents the case group (effect) (A) Affected pathways in *Syngap1*$^{+/-}$ mouse (*Syngap1*$^{+/-}$: Saline Vs WT: Saline). (B) Effect of CSP-TTK21 treatment (*Syngap1*$^{+/-}$: CSP-TTK21 Vs *Syngap1*$^{+/-}$: Saline). (C) Direct effect of CSP-TTK21 (*Syngap1*$^{+/-}$: CSP-TTK21 Vs *Syngap1*$^{+/-}$: CSP).

S10.

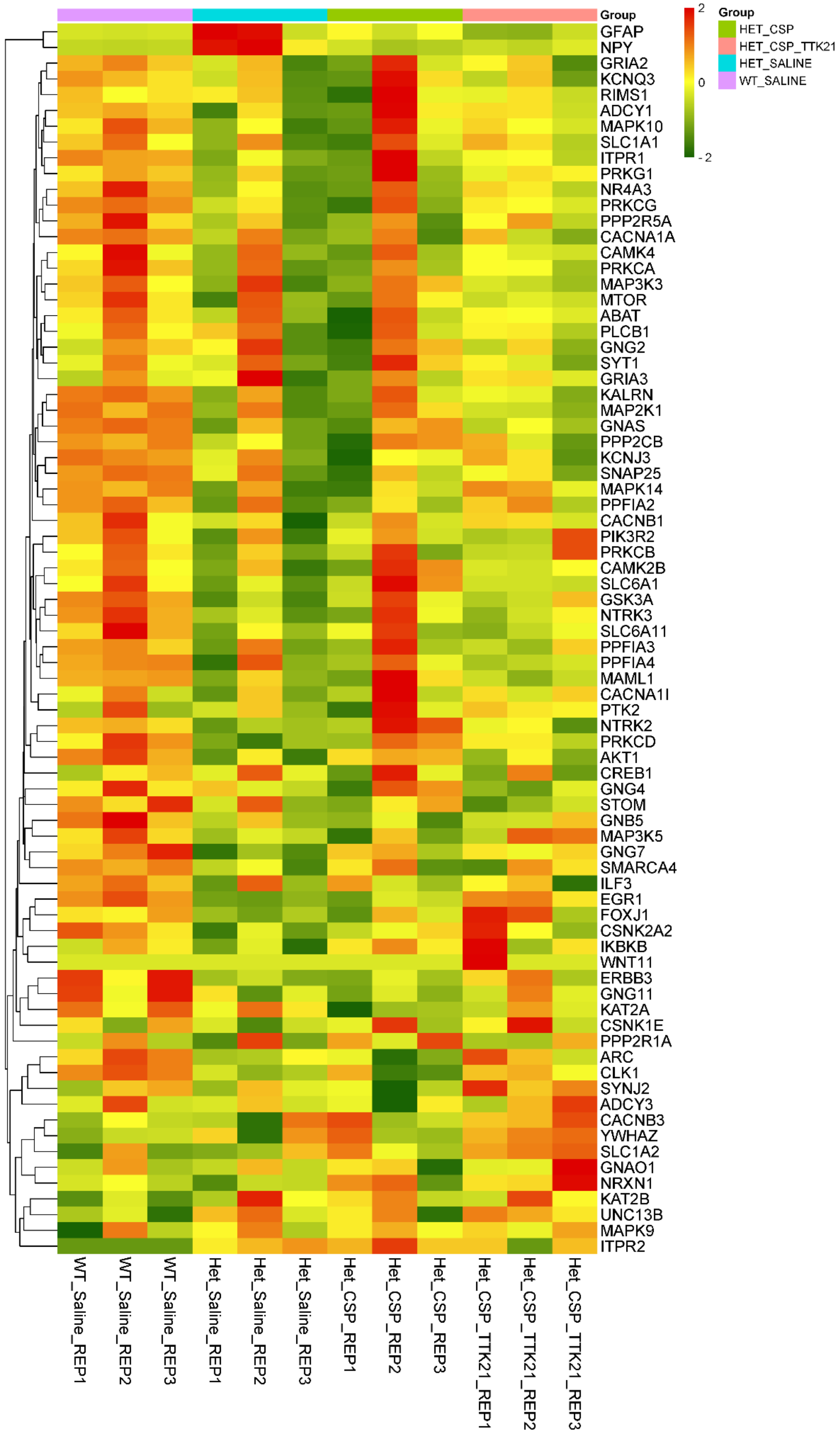
Group
Group
HET_CSP
HET_CSP_TTK21
HET_SALINE
WT_SALINE
2
0
-2
GFAP
NPY
GRIA2
KCNQ3
RIMS1
ADCY1
MAPK10
SLC1A1
ITPR1
PRKG1
NR4A3
PRKCG
PPP2R5A
CACNA1A
CAMK4
PRKCA
MAP3K3
MTOR
ABAT
PLCB1
GNG2
SYT1
GRIA3
KALRN
MAP2K1
GNAS
PPP2CB
KCNJ3
SNAP25
MAPK14
PPFIA2
CACNB1
PIK3R2
PRKCB
CAMK2B
SLC6A1
GSK3A
NTRK3
SLC6A11
PPFIA3
PPFIA4
MAML1
CACNA1I
PTK2
NTRK2
PRKCD
AKT1
CREB1
GNG4
STOM
GNB5
MAP3K5
GNG7
SMARCA4
ILF3
EGR1
FOXJ1
CSNK2A2
IKBKB
WNT11
ERBB3
GNG11
KAT2A
CSNK1E
PPP2R1A
ARC
CLK1
SYNJ2
ADCY3
CACNB3
YWHAZ
SLC1A2
GNAO1
NRXN1
KAT2B
UNC13B
MAPK9
ITPR2
WT_Saline_REP1
WT_Saline_REP2
WT_Saline_REP3
Het_Saline_REP1
Het_Saline_REP2
Het_Saline_REP3
Het_CSP_REP1
Het_CSP_REP2
Het_CSP_REP3
Het_CSP_TTK21_REP1
Het_CSP_TTK21_REP2
Het_CSP_TTK21_REP3

Fig. S10. **Heatmap showing the expression levels of topmost genes that were rescued upon CSP-TTK21 treatment (3 mice/group). Red represents up-regulation and green represents down-regulation. WT- Wild type and Het- *Syngap1*$^{+/-}$ mice.**

Table S1. Primer list

Gene names	Forward primer	Reverse primer
**Adcy1**	TCGTGTCCTATGCCTTGCTG	CTCTGTCAAGATCCGCACGA
**Arc**	TGAGACCAGTTCCACTGCTG	CTCCAGGGTCTCCCTAGTCC
**Egr1**	TGCACCCACCTTTCCTACTC	AGGTCTCCCTGTTGTTGTGG
**Gapdh**	TTGTGATGGGTGTGAACCACGAGA	GAGCCCTTCCACAATGCCAAAGT
**Kcnq3**	ACAGGTTGGATCTGGTGGCA	TACCACGAGGATTAGAAAGGCG
**Foxj1**	TGTCGGCCATCTACAAGTGG	CTTCTCCCGAGGCACTTTGA
**Nr4a3**	ACGCCGAAACCGATGTCAGTAC	CTCCTGTTGTAGTGGGCTCTTTG
**Ntrk3**	GCATTGAGTTTGTGGTGCGT	GTAGGGCTCGCATCAGACTC
**Gng7**	CTGACTCTCAGAACCTGGCTTG	TTCTCCTTGCCAGTGTAGCTGG
**Npy**	GATACTACTCCGCTCTGCGAC	CATCACCACATGGAAGGGTCT
**Erbb3**	GACACAGATTGCTTCGCCTG	AGGACAAGCCCTGACACAAA
**Itpr1**	CGGATGCCAGGAGGAAATGT	CTCAGGGGTGGACTTGGTTC
**Kalrn1**	GGTATCTCCGCTTGCTTCGG	TCGATGATGACGGTGAAGCC
